# *APC* gene 3′UTR SNPs and interactions with environmental factors are correlated with risk of colorectal cancer in Chinese Han population

**DOI:** 10.1042/BSR20192429

**Published:** 2020-03-20

**Authors:** Rongbiao Ying, Zhiping Wei, Yuxian Mei, Shasha Chen, Liming Zhu

**Affiliations:** 1 Department of Surgical Oncology, Taizhou Cancer Hospital, No. 50 Zhenxin Road, Wenling, Zhejiang, China; 2 Department of Urology, Wenling Hospital of Traditional Chinese Medicine, No. 21, Mingyuan North Road, Wenling, Zhejiang, China; 3 Department of Traditional Chinese Medicine, Taizhou Cancer Hospital, No. 50 Zhenxin Road, Wenling, Zhejiang, China; 4 Institute of Cancer and Basic Medicine (ICBM), Chinese Academy of Sciences, Hangzhou, Zhejiang, China; 5 Department of Medical Oncology, Cancer Hospital of the University of Chinese Academy of Sciences, Hangzhou, Zhejiang, China; 6 Department of Medical Oncology, Zhejiang Cancer Hospital, Hangzhou, Zhejiang, China

**Keywords:** adenomatous polyposis coli, colorectal cancer, gene-environment interaction, single nucleotide polymorphism

## Abstract

**Objective:** To study the correlation between *adenomatous polyposis coli* (*APC*) gene 3′ untranslated region (UTR) single nucleotide polymorphisms (SNPs) and their interactions with environmental factors and the risk of colorectal cancer (CRC) in a Chinese Han population.

**Methods:** Genotypes of *APC* gene 3′UTR rs1804197, rs41116, rs448475, and rs397768 loci in 340 Chinese Han patients with CRC and 340 healthy controls were analyzed. All patients with CRC were analyzed for progression-free survival (PFS) during a 3-year follow-up.

**Results:** The risk of CRC in subjects carrying the *APC* gene rs1804197 A allele was 2.95-times higher than for the C allele carriers. The interactions of the rs1804197 SNP with body mass index (BMI) and smoking were associated with the risk of CRC. The risk of CRC in the *APC* gene rs397768 G allele carriers was 1.68-times higher than in the A allele carriers. The interaction between the rs397768 locus SNP and gender was also associated with the risk of CRC. The 3-year PFS of patients with *APC* gene rs1804197 AA genotype, CA genotype, and CC genotype CRC decreased in this order, with significant difference. In addition, the 3-year PFS of rs397768 locus GG genotype, AG genotype, and AA genotype CRC patients decreased in this order, and the difference was significant.

**Conclusion:** The rs1804197 locus in the 3′UTR region of the *APC* gene and its interactions with BMI and smoking are associated with the risk of CRC in a Chinese Han population. In addition, the interaction between rs397768 locus SNP and gender is related to the risk of CRC.

## Introduction

Colorectal cancer (CRC) is a common malignant tumor of the digestive tract, and its incidence in China has shown an increasing trend in recent years [1]. With the development of tumor molecular biology, the study and understanding of CRC have entered a new stage. CRC is a systemic kind of disease involving multiple stages, signaling pathways, and pathology-related genes, and is characterized by an ethnic distribution, familial aggregation, and genetic defects [2–4].

Owing to biological diversity, that is, human heterogeneity and the multiple steps leading up to carcinogenesis, individuals have different sensitivities to carcinogen exposure [5]. Genetic polymorphism is a critical reason for differences in response to environmental factors. Single nucleotide polymorphism (SNP) is an important part of functional genomics, being one of the genetic bases of individual differences, and analysis of it is important for studying the genetic features of tumors. For example, *XPC* gene polymorphisms have been linked to susceptibility to CRC [6,7]. Thus, properly identifying genes and their allelic variants, and exploring the interactions between environmental factors and susceptibility genes is extremely important for defining higher risk populations, assessing the risk of disease and developing an effective earlier warning system.

The *adenomatous polyposis coli* (*APC*) gene is one of the genes most closely related to CRC. This tumor suppressor gene was discovered by Herrera et al. [8]. Its product, APC protein, is mainly involved in Wnt/β-catenin signaling. It forms a complex with axin and glycogen synthase kinase-3β (GSK-3β), which ensures the normal regulation of the Wnt/β-catenin signaling pathway in cell differentiation, proliferation, polarity, and migration [9]. There are diverse variants of the *APC* gene in patients with CRC [10,11]. Dysfunction of this gene is believed to be related to the development of CRC, by affecting not only the proliferation and differentiation of epithelial cells, but also the adhesion and migration [12,13].

In the present study, the rs1804197, rs41116, rs448475, and rs397768 loci in the 3′ untranslated region (3′UTR) region of the *APC* gene were selected. The minor allele frequency (MAF) of the rs1804197 locus in the southern Chinese Han population is 0.1095. A previous study found that rs1804197 SNP was associated with autism spectrum disorder (ASD) [14]. The MAF of the rs41116 locus is 0.3190. A number of studies have focused on this locus [15–17]; however, no study has yet confirmed that it is related to CRC risk. The MAF of rs448475 is 0.3190. It was reported that SNP in this locus is associated with non-syndromic cleft lip with or without cleft palate (NSCL±P), which may be related to the binding of microRNA-617 [18]. The MAF of the rs397768 locus is 0.2143, which was previously shown to potentially be associated with breast cancer [17]. The above four SNP loci are located in the 3′UTR region of the *APC* gene, which can bind to microRNA, degrade mRNA, or inhibit mRNA translation to regulate *APC* expression. The aim of the current study was to analyze the association between rs1804197, rs41116, rs448475, and rs397768 SNPs in the 3′UTR region of the *APC* gene and the risk of CRC, as well as to explore the effect of their interactions with environmental factors on CRC.

In summary, through a case–control study, we found that the rs1804197 locus in the 3′UTR region of the *APC* gene and its interactions with body mass index (BMI) and smoking were associated with the risk of CRC in the Chinese Han population. Moreover, the SNP of the rs397768 locus and its interaction with gender were related to the risk of CRC.

## Materials and methods

### Basic information on the participants

A total of 340 Chinese Han patients with CRC were recruited from Taizhou Cancer Hospital and Zhejiang Cancer Hospital between February 2014 and January 2016, including 177 males and 163 females, aged 39–85 years. All patients were identified as having CRC by pathological diagnosis. The tumor-node-metastasis (TNM) staging was established with reference to the International Union Against Cancer (UICC) cancer staging criteria [19], with 54 cases in stage I, 62 cases in stage II, 126 cases in stage III, and 98 cases in stage IV. Another cohort of 340 healthy individuals without CRC was enrolled as a control group, with 181 males and 159 females, aged 41–83 years. There were no significant differences between the two groups in age, gender, BMI, smoking, drinking status, and other factors (*P*>0.05), as shown in Table 1. The control subjects did not have a history of tumors. Informed consent was signed by all CRC patients and control subjects who participated in the present study. The study was approved by the Medical Ethics Committee of Taizhou Cancer Hospital and Zhejiang Cancer Hospital. The recruitment was performed in accordance with the principles of the World Medical Association’s Declaration of Helsinki.

**Table 1:** Comparison of general characteristics between CRC patients and healthy controls

	CRC (*n*=340)	Control (*n*=340)	*P*
Age (years, mean ± SD)	62.05 ± 7.97	62.42 ± 8.44	0.56
Gender (*n*, %)			
Male	177 (52.06%)	181 (53.24%)	0.76
Female	163 (47.94%)	159 (46.76%)	
BMI (kg/m^2^, mean ± SD)	25.92 ± 2.21	25.84 ± 2.35	0.65
Smoking (*n*, %)			
Yes	85 (25.00%)	78 (22.94%)	0.53
No	255 (75.00%)	262 (77.06%)	
Alcohol (*n*, %)			
Yes	90 (26.47%)	97 (28.53%)	0.55
No	250 (73.53%)	243 (71.47%)	

The criteria for smoking: smoking of one or more cigarettes per day; the criteria for drinking alcohol: drinking more than 50 g of alcohol per day. Abbreviation: SD, standard deviation.

### Genotype analysis

To determine the genotypes of *APC* gene 3′UTR SNPs, 5 ml of peripheral venous blood was collected from all participants, and the genomic DNA was extracted using a DNA extraction kit (TIANGEN Biotech Co. Ltd., Beijing, China), and stored in a freezer at −70°C before testing. The genotype was analyzed by PCR/Sanger sequencing. The rs1804197 locus primers were: 5′-GAG GGT TTT TGT TCT GGA AGC C-3′ (forward); 5′-CCA TCA AGA GTG CCT CCC AA-3′ (reverse). The rs41116 locus primers were: 5′-CAT TCC ATG CGT TGG CAC TT-3′ (forward); 5′-AGT CTG TGC TAG GCT GCT TG-3′ (reverse). The rs448475 locus primers were: 5′-TCC CTG CCT GTT AAG GAA ACT-3′ (forward); 5′-CCT CCA CTG TAT AAG GGG ACA C-3′ (reverse). The rs397768 locus primers were: 5′-ACA CTC TGT ATT TGG GGA GGG-3′ (forward); 5′-TCA AGG CAC CAG GTA GGT GT-3′ (reverse). The PCR mixture contained 20 ng of genomic DNA, 2 μl of 10× PCR buffer, 20 pmol of each primer, 0.5 U of Taq DNA polymerase, and 1.6 μl of 2.5 mmol/l dNTP. The PCR was carried out under the following conditions: pre-denaturation at 94°C for 1 min; then denaturation at 94°C for 30 s, annealing at 56°C for 30 s, and extension at 72°C for 1 min, for a total of 35 cycles; followed by extension at 72°C for 10 min. Sanger sequencing was used to determine the sequence of the PCR products, and the SNP genotype was determined by comparison with the sequence in an online database (https://www.ncbi.nlm.nih.gov/snp/).

### Clinical follow-up

All patients were followed up to 3 years. The first patient was followed up from February 2014 to February 2017, while the last one was followed up until January 2019. All patients’ progression-free survival (PFS) was recorded.

### Statistical analyses

Continuous variables were expressed as mean ± SD and statistical analyses were conducted using an independent *t* test. The categorical variables were expressed as a percentage [*n* (%)] and the statistical analysis was performed using the *χ*^2^ test. The genotype frequency was analyzed for Hardy–Weinberg equilibrium by the *χ*^2^ test. The correlation between SNPs in the 3′UTR region of the *APC* gene and the risk of CRC was determined based on the distribution of allele frequencies and genetic models (additive, dominant, and recessive models). The odds ratio (OR) and 95% confidence interval (CI) were calculated in an unconditional logistic regression analysis, adjusted for age, gender, BMI, smoking, drinking, and other factors. Statistical analyses in the present study were performed using SPSS22.0 software (IBM, Chicago, IL). Multifactor dimensionality reduction (MDR) analysis was used to analyze the interaction between the rs1804197 and rs397768 loci and age, gender, BMI, smoking, drinking, and other factors. All tests were two-tailed and *P*<0.05 was considered statistically significant.

## Results

### Correlation between SNP in the 3′UTR region of the APC gene and the risk of CRC

The genotype frequency distributions of the 3′UTR region of the *APC* gene rs1804197, rs41116, rs448475, and rs397768 loci were consistent with Hardy–Weinberg equilibrium (*P*>0.05), as shown in Table 2. Taking the CC genotype of the rs1804197 locus as a reference, the CA and AA genotype frequencies of CRC patients were significantly higher than those in the control group (*P*<0.05). The risk of CRC was not significantly increased in the additive model; however, it was significantly enhanced in both the dominant and recessive models (*P*<0.001). The risk of CRC in subjects with the A allele was 2.95-times higher than in C allele carriers (95% CI: 2.14–4.05, *P*<0.001). There were no significant differences in the genotype and allele frequencies of rs41116 and rs448475 loci between CRC patients and controls (*P*>0.05). Taking the AA genotype of the rs397768 locus as a reference, the difference in the AG genotype frequency between CRC patients and the control group was not statistically significant (*P*>0.05), while the frequency of the GG genotype was significantly higher in CRC patients than in the control group (*P*<0.001). The risk of CRC was not increased in the additive model (*P*>0.05); however, it was significantly increased in the dominant and recessive models (*P*<0.05). The risk of CRC in the G allele carriers was 1.68-times higher than that in the A allele carriers (95% CI: 1.30–2.18, *P*<0.001).

**Table 2:** Correlation between genotype and allele frequency of *APC* gene 3′UTR region SNPs and the risk of CRC

	CRC (*n*=340)	Control (*n*=340)	HWE *P*	OR (95% CI)*	*P*
**rs1804197**					
CC	226 (66.47%)	284 (83.53%)	0.13	1.00 (Reference)	
CA	75 (22.06%)	51 (15.00%)		1.85 (1.24–2.75)	**0.002**
AA	39 (11.47%)	5 (1.47%)		9.80 (3.80–25.28)	**<0.001**
Additive				1.26 (0.99–1.58)	0.06
Dominant				2.56 (1.78–3.68)	**<0.001**
Recessive				8.68 (3.38–22.31)	**<0.001**
C	527 (77.50%)	619 (91.03%)		1.00 (Reference)	
A	153 (22.50%)	61 (8.97%)		2.95 (2.14–4.05)	<0.001
**rs41116**					
CC	191 (56.18%)	186 (54.71%)	0.16	1.00 (Reference)	
CT	118 (34.71%)	124 (36.47%)		0.93 (0.67–1.28)	0.64
TT	31 (9.12%)	30 (8.82%)		1.01 (0.59–1.73)	0.98
Additive				0.97 (0.76–1.25)	0.84
Dominant				0.94 (0.70–1.28)	0.70
Recessive				1.04 (0.61–1.75)	0.89
C	500 (73.53%)	496 (72.94%)		1.00 (Reference)	
T	180 (26.47%)	184 (27.06%)		0.97 (0.76–1.23)	0.81
**rs448475**					
GG	177 (52.06%)	167 (49.12%)	0.18	1.00 (Reference)	
GC	129 (37.94%)	135 (39.71%)		0.90 (0.65–1.24)	0.53
CC	34 (10.00%)	38 (11.18%)		0.84 (0.51–1.40)	0.51
Additive				0.94 (0.73–1.22)	0.66
Dominant				0.89 (0.66–1.20)	0.44
Recessive				0.88 (0.54–1.44)	0.62
G	483 (71.03%)	469 (68.97%)		1.00 (Reference)	
C	197 (28.97%)	211 (31.03%)		0.91 (0.72–1.14)	0.41
rs397768					
AA	201 (59.12%)	228 (67.06%)	0.58	1.00 (Reference)	
AG	91 (26.76%)	99 (29.12%)		1.04 (0.74–1.47)	0.81
GG	48 (14.12%)	13 (3.82%)		4.19 (2.21–7.96)	< 0.001
Additive				1.13 (0.89–1.45)	0.31
Dominant				1.41 (1.03–1.93)	0.03
Recessive				4.14 (2.20–7.79)	< 0.001
A	493 (72.50%)	555 (81.62%)		1.00 (Reference)	
G	187 (27.50%)	125 (18.38%)		1.68 (1.30–2.18)	< 0.001

Abbreviation: HWE, Hardy–Weinberg equilibrium.

* Adjusted for factors such as age, gender, BMI, smoking, and drinking.

### Stratified analysis of the correlation between *APC* gene rs1804197 SNP and the risk of CRC

A stratified analysis of age, gender, BMI, smoking, and alcohol drinking status revealed that the risk of CRC was significantly increased in the *APC* gene rs1804197 A allele carriers at both ≥60 and <60 years of age (*P*<0.05). The risk of CRC was significantly increased in both males and females carrying the *APC* gene rs1804197 A allele (*P*<0.05). Besides, only in patients with BMI ≥ 24 kg/m^2^, the risk of CRC in the *APC* gene rs1804197 A allele carriers was significantly increased (*P*<0.001), while in the population with BMI < 24 kg/m^2^, the risk of CRC in the *APC* gene rs1804197 A allele carriers was decreased (*P*>0.05). In non-smokers, the risk of CRC was significantly higher in subjects carrying the *APC* gene rs1804197 A allele (*P*<0.001), whereas in smokers, the subjects carrying the *APC* gene rs1804197 A allele were not at increased risk for CRC (*P*>0.05). In addition, in both drinking and non-drinking subjects, the risk of CRC in the *APC* gene rs1804197 A allele carriers was significantly increased (*P*<0.001), as shown in Table 3.

**Table 3:** Stratified analysis of the correlation between *APC* gene rs1804197 SNP and the risk of CRC

	CRC (*n*=340)	Control (*n*=340)	OR (95% CI)*	*P*
Age (years)				
≥60				
CC	91 (62.76%)	111 (79.86%)	1.00 (Reference)	
CA/AA	54 (37.24%)	28 (20.14%)	2.35 (1.38–4.01)	**0.001**
<60				
CC	135 (69.23%)	173 (86.07%)	1.00 (Reference)	
CA/AA	60 (30.77%)	28 (13.93%)	2.75 (1.66–4.54)	**<0.001**
Gender				
Male				
CC	118 (66.67%)	145 (80.11%)	1.00 (Reference)	
CA/AA	59 (33.33%)	36 (19.89%)	2.01 (1.25–3.26)	**0.004**
Female				
CC	108 (66.26%)	139 (87.42%)	1.00 (Reference)	
CA/AA	55 (33.74%)	20 (12.58%)	3.54 (2.00–6.26)	**<0.001**
BMI (kg/m^2^)				
≥24				
CC	180 (63.60%)	229 (81.49%)	1.00 (Reference)	
CA/AA	103 (36.40%)	52 (18.51%)	2.52 (1.71–3.71)	**<0.001**
<24				
CC	46 (80.70%)	55 (93.22%)	1.00 (reference)	
CA/AA	11 (19.30%)	4 (6.78%)	3.29 (0.98–11.02)	0.08
Smoking				
Yes				
CC	57 (67.06%)	62 (79.49%)	1.00 (reference)	
CA/AA	28 (32.94%)	16 (20.51%)	1.90 (0.93-3.88)	0.07
No				
CC	169 (66.27%)	222 (84.73%)	1.00 (Reference)	
CA/AA	86 (33.73%)	40 (15.27%)	2.82 (1.85–4.32)	**<0.001**
Alcohol				
Yes				
CC	59 (65.56%)	85 (87.63%)	1.00 (Reference)	
CA/AA	31 (34.44%)	12 (12.37%)	3.72 (1.77–7.84)	**<0.001**
No				
CC	167 (66.80%)	199 (81.89%)	1.00 (Reference)	
CA/AA	83 (33.20%)	44 (18.11%)	2.25 (1.48–3.42)	**<0.001**

* Adjusted for factors such as age, gender, BMI, smoking, and drinking.

### Stratified analysis of the correlation between *APC* gene rs397768 SNP and the risk of CRC

Further, analysis stratified by age, gender, BMI, smoking, and drinking status showed that the risk of CRC was not significantly increased in the *APC* gene rs397768 G allele carriers in subjects aged both ≥60 and <60 years (*P*<0.05). The risk of CRC was significantly increased in females carrying the G allele of the *APC* gene rs397768 locus (*P*<0.05), while males carrying the *APC* gene rs397768 G allele were not at risk for CRC (*P*>0.05). The subjects carrying the G allele in the rs397768 locus in the *APC* gene had no risk of CRC at both BMI ≥ 24 kg/m^2^ and BMI < 24 kg/m^2^ (*P*>0.05). Moreover, in smokers and non-smokers, the risk of CRC was significantly higher in carriers of the G allele of the rs397768 locus of the *APC* gene (*p* < 0.05), and in drinking and non-drinking subjects, the carriers of the G allele of the rs397768 locus in the *APC* gene did not have an increased risk of CRC (*p* > 0.05), as shown in detail in Table 4.

**Table 4:** Stratified analysis of the correlation between *APC* gene rs397768 SNP and the risk of CRC

	CRC (*n*=340)	Control (*n*=340)	OR (95% CI)*	*P*
Age				
≥60				
AA	81 (55.86%)	93 (66.91%)	1.00 (Reference)	
AG/GG	64 (44.14%)	46 (33.09%)	1.60 (0.99–2.59)	0.07
<60				
AA	120 (61.54%)	135 (67.16%)	1.00 (Reference)	
AG/GG	75 (38.46%)	66 (32.84%)	1.28 (0.85–1.93)	0.24
Gender				
Male				
AA	109 (61.58%)	112 (61.88%)	1.00 (Reference)	
AG/GG	68 (38.42%)	69 (38.12%)	1.01 (0.66–1.55)	0.95
Female				
AA	92 (56.44%)	116 (72.96%)	1.00 (Reference)	
AG/GG	71 (43.56%)	43 (27.04%)	2.08 (1.31–3.32)	**0.002**
BMI (kg/m^2^)				
≥24				
AA	164 (57.95%)	184 (65.48%)	1.00 (Reference)	
AG/GG	119 (42.05%)	97 (34.52%)	1.38 (0.98–1.94)	0.08
<24				
AA	37 (64.91%)	44 (74.58%)	1.00 (Reference)	
AG/GG	20 (35.09%)	15 (25.42%)	1.59 (0.71–3.53)	0.26
Smoking				
Yes				
AA	64 (75.29%)	42 (53.85%)	1.00 (Reference)	
AG/GG	21 (24.71%)	36 (46.15%)	0.38 (0.20–0.74)	**0.004**
No				
AA	137 (53.73%)	186 (70.99%)	1.00 (Reference)	
AG/GG	118 (46.27%)	76 (29.01%)	2.11 (1.47–3.03)	**< 0.001**
Alcohol				
Yes				
AA	47 (52.22%)	58 (59.79%)	1.00 (Reference)	
AG/GG	43 (47.78%)	39 (40.21%)	1.36 (0.76–2.43)	0.30
No				
AA	154 (61.60%)	170 (69.96%)	1.00 (Reference)	
AG/GG	96 (38.40%)	73 (30.04%)	1.45 (0.99–2.11)	0.06

* Adjusted for factors such as age, gender, BMI, smoking, and drinking.

### Haplotype analysis

**Figure 1:**
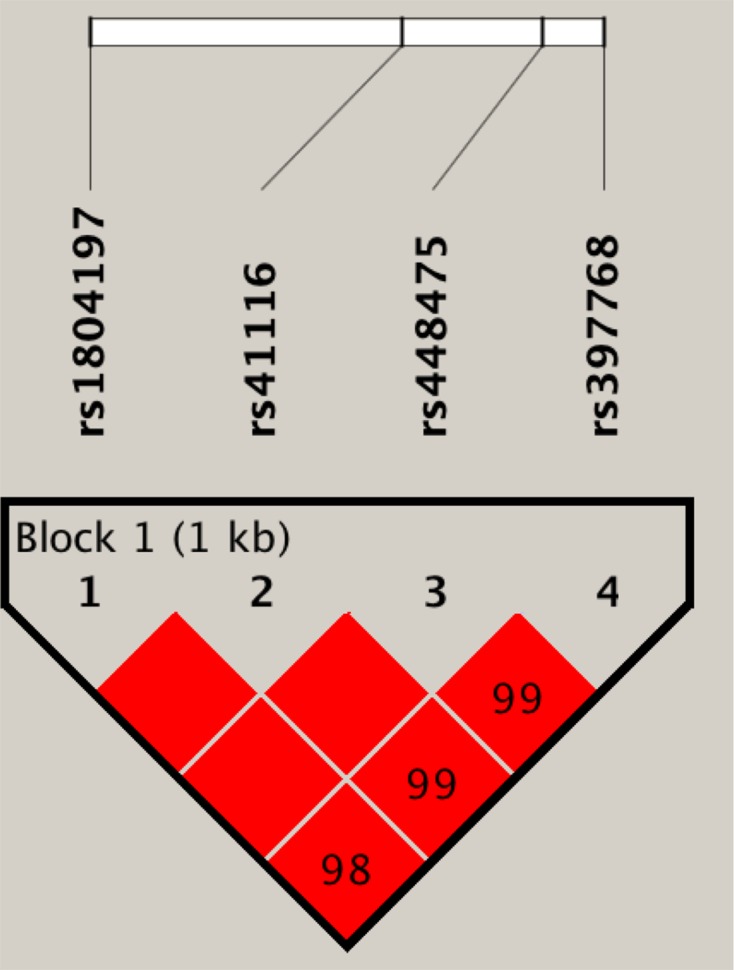
Linkage disequilibrium map of the *APC* gene rs1804197, rs41116, rs448475, and rs397768 SNP loci

Four haplotypes were found in the *APC* gene rs1804197, rs41116, rs448475, and rs397768 loci; namely, ACGG, CTCG, CTCA, and ATCA, respectively (Figure 1). Analysis of the results showed that the risk of CRC in carriers of the haplotype ACGG was significantly increased by 3.24-times (95% CI: 1.58–6.25, *P*<0.001), as shown in Table 5.

**Table 5:** Correlation between haplotypes of SNP loci in the 3′UTR region of the *APC* gene and the risk of CRC

rs1804197/rs41116/rs448475/rs397768	CRC (*n*=340)	Control (*n*=340)	OR (95% CI)	*P*
ACGG	150 (44.12%)	90 (26.47%)	3.24 (1.58–6.25)	<0.001
CTCG	103 (30.29%)	124 (36.47%)	0.85 (0.55–1.24)	0.09
CTCA	47 (13.82%)	61 (17.94%)	0.91 (0.87–1.54)	0.44
ATCA	40 (11.76%)	65 (19.12%)	0.77 (0.62–1.03)	0.31

The false positive report rates (FPRPs) at different levels of prior probability are shown in Table 6. In those aged ≥60 or <60 years, males, females, those with BMI ≥ 24 kg/m^2^, with a smoking history, no smoking history, and no drinking history, there were significant correlations between the susceptibility to CRC and the rs1804197 CA/AA genotype, with an FFRP below 0.2 when the prior probability was 0.1. In females and those without a history of smoking, carrying the rs397768 AG/GG genotype was significantly associated with susceptibility to CRC. When the prior probability was 0.1, the FFRP was less than 0.2. In those with a history of smoking, the FFRP value of the correlation between carrying the rs397768 AG/GG genotype and the susceptibility to CRC was greater than 0.2, indicating that the sample size might be small and the results might be biased. As such, this needs further studies in large samples.

**Table 6:** FPRP value of correlation between *APC* gene SNPs and CRC susceptibility

Genotype	OR (95% CI)	Statistical power	Prior probability		
			0.1	0.01	0.001
rs1804197 CA vs CC	1.85 (1.24–2.75)	0.934	0.1957	0.7279	0.9643
rs1804197 AA vs CC	9.80 (3.80–25.28)	0.972	0.0439	0.3356	0.8360
rs397768 AA vs GG	4.19 (2.21–7.96)	0.890	0.0970	0.5416	0.9226
rs1804197 CA/AA vs CC					
Age ≥60	2.35 (1.38–4.01)	0.913	0.1607	0.6781	0.9551
Age <60	2.75 (1.66–4.54)	0.871	0.1406	0.6429	0.9478
Male	2.01 (1.25–3.26)	0.659	0.1829	0.7112	0.9613
Female	3.54 (2.00–6.26)	0.889	0.1128	0.5830	0.9338
BMI ≥24 kg/m^2^	2.52 (1.71–3.71)	0.932	0.1515	0.6627	0.9520
Never smoking	2.82 (1.85–4.32)	0.954	0.1376	0.6371	0.9466
Ever drinking	3.72 (1.77–7.84)	0.881	0.1079	0.5709	0.9307
Never drinking	2.25 (1.48–3.42)	0.923	0.1667	0.6875	0.9569
rs397768 AG/GG vs AA					
Female	2.08 (1.31–3.32)	0.893	0.1779	0.7041	0.9600
Ever smoking	0.38 (0.20–0.74)	0.342	0.5422	0.9287	0.9924
Never smoking	2.11 (1.47–3.03)	0.931	0.1758	0.7011	0.9595

### MDR analysis of the interaction between genes and environmental factors

**Figure 2:**
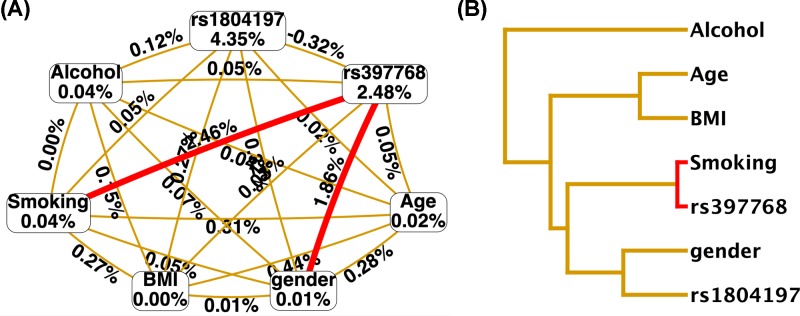
MDR analysis of interactions between *APC* gene rs1804197 and rs397768 loci SNPs and the subject’s age, gender, BMI, smoking, drinking and other environmental factors (**A**) Circle graph, the percentage at the bottom of each polymorphism represents the correlation with CRC, with a larger value representing a greater contribution to CRC. The percentage on each line represents the interaction between the two factors, with a larger value representing a stronger interaction. (**B**) Tree graph. Red represents the stronger interaction between the two factors and a closer location between two factors represents their stronger interaction.

MDR was used to analyze the interaction between *APC* gene SNPs rs1804197 and rs397768 and environmental factors such as age, sex, BMI, smoking, and drinking status. The results showed a robust interaction between rs397768 and smoking, followed by the interaction between rs397768 and gender, as shown in Figure 2.

### Association of the *APC* gene 3′UTR SNPs with the PFS of patients with CRC

**Figure 3:**
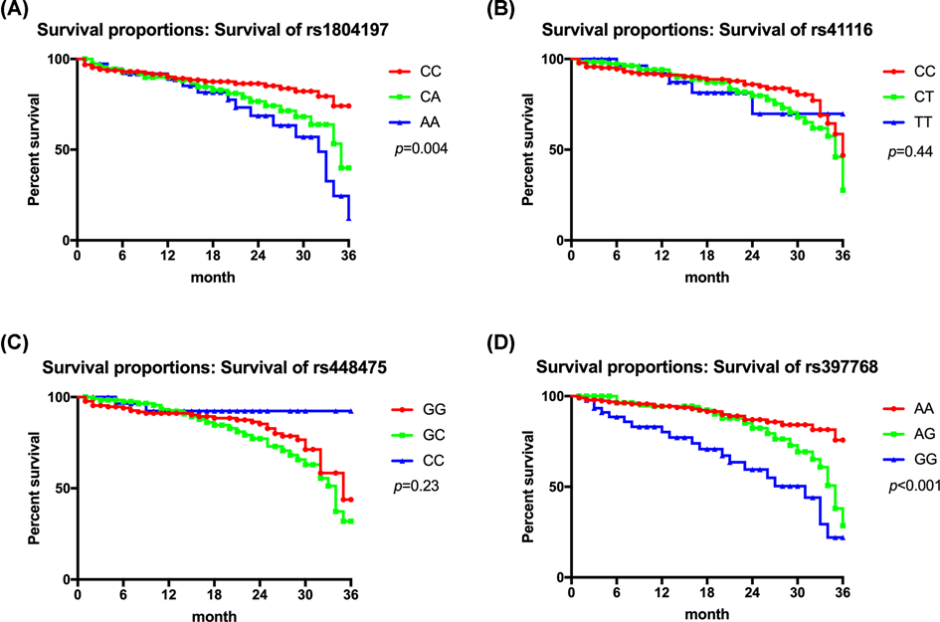
Correlation between the *APC* gene 3′UTR region SNPs and PFS of CRC patients (**A**) Three-year PFS of CRC patients with different genotypes of the rs1804197 locus. (**B**) Three-year PFS of CRC patients with different genotypes of the rs41116 locus. (**C**) Three-year PFS of CRC patients with different genotypes of the rs448475 locus. (**D**) Three-year PFS of CRC patients with different genotypes of the rs397768 locus.

After 3 years of follow-up, we found that the PFS of CRC patients significantly differed among subjects with the *APC* gene rs1804197 AA, CA, and CC genotypes, in decreasing order (*P*=0.004; Figure 3A). However, there was no significant difference in the 3-year PFS between different genotypes in the rs41116 and rs448475 loci of CRC patients (*P*>0.05; Figure 3B,C). The 3-year PFS of CRC patients differed significantly among subjects with rs397768 locus GG, AG, and AA genotypes, in decreasing order (*P*<0.001; Figure 3D).

## Discussion

With increasing progress in molecular biology, the study of the etiology of CRC has advanced from environmental factors to environmental–genetic interactions, and gradually encroached on analyses at molecular level [20,21]. By investigating the molecular mechanisms behind the formation of tumors, a clearer understanding of the relationship among environment, genetics, genes, and CRC has been established. This advance in our knowledge has changed the treatment of CRC from surgery-based approaches to a comprehensive toolkit of surgery, radiotherapy, chemotherapy, and targeted therapy [22].

After extensive studies of the pathogenesis, it was gradually realized that the progression of CRC is a continuous multistage process. Under the influence of internal and external environmental factors such as the continuous accumulation of genetic changes and disruption of the normal regulation of cell division, apoptosis, and tissue self-stability, survival advantages develop, eventually leading to a malignant tumor [23,24]. Previous studies found that SNPs are one of the main causes of differences in tumor susceptibility [25]. Genetic material is impaired by a variety of factors, leading to reduced genomic stability; in particular, the interaction between genetic and environmental factors has been recognized as a factor contributing to a variety of tumors [26].

The *APC* gene is a tumor suppressor gene, and the product of its expression is an important component of the Wnt signaling pathway, which plays a critical regulatory role in cell growth, apoptosis, and signal transmission [27]. Inactivation of the *APC* gene results in disruption of the degradation of β-catenin, leading to the accumulation of free β-catenin in the cytoplasm and its translocation into the nucleus. This in turn activates Tcf/Lef, causing abnormal transcription of the *c-myc, c-jun*, and *cyclin D1* genes, which eventually causes cancers to develop [28]. Changes in *APC* protein expression levels may be associated with the development of CRC [29]. In the present study, the selected SNP loci are located in the 3′UTR region of the *APC* gene, which is the region where microRNAs bind to the *APC* gene. The regulation of *APC* gene expression by microRNAs may be related to the efficiency of binding of microRNA to the *APC* gene 3′UTR region.

The results of the present study show that the risk of CRC in subjects carrying the A allele of the rs1804197 locus in the *APC* gene was 2.95-times higher than in carriers of the C allele. A previous study found that the rs1804197 SNP is associated with ASD [14], and suggested that this SNP may be related to the expression level of APC protein. Our further analyses showed that the interactions of the rs1804197 SNP with BMI and smoking were associated with the risk of CRC, suggesting that obesity and smoking may have a certain impact on the risk of CRC in patients with different rs1804197 genotypes. In obese patients, the rs1804197 A allele carriers have a higher risk of CRC, whereas in non-smokers, the rs1804197 A allele carriers have a higher risk of it. Interestingly, there is no cumulative effect between smoking and the rs1804197 SNP; however, there is a cumulative effect between non-smoking and the rs1804197 SNP. We speculate that smoking may also be a risk factor for CRC and confers a higher risk of CRC regardless of which rs1804197 locus genotype is carried.

We also found that the risk of CRC in *APC* gene rs397768 G allele carriers was 1.68-times higher than in A allele carriers, after adjustment for age, gender, BMI, smoking, drinking, and other factors. Further analyses revealed that the interaction between rs397768 locus SNP and gender was associated with the risk of CRC, as it was shown that only in female subjects was the risk of CRC significantly increased in the *APC* gene rs397768 G allele carriers. We suspect that this may be related to bad habits. Specifically, men are more likely to be smokers, drinkers, and have bad eating habits than women. Therefore, the risk of CRC is relatively high in males, regardless of the alleles of rs397768. It was also shown previously that men are more susceptible to CRC than women [30].

We further found that the *APC* gene rs1804197 SNP was associated with 3-year PFS in patients with CRC, and the 3-year PFS differed among patients with AA, CA, and CC genotypes, in descending order. The *APC* gene rs397768 locus SNP was also associated with 3-year PFS in patients with CRC, and the 3-year PFS varied among CRC patients with GG, AG, and AA genotypes, in descending order. We considered that the reason for this may be that *APC* gene rs1804197 and rs397768 SNPs are related to the expression level of APC. Low expression or inactivation of APC is one of the causes of cell hyperproliferation, which may eventually lead to a reduction in 3-year PFS in CRC patients. However, further studies are needed to confirm this.

The present study has several limitations. First, it was not clear which microRNA binds to the rs1804197 and rs397768 loci in the 3′UTR region of the *APC* gene, and therefore there is no direct evidence to support the correlation between the regulation of APC expression by microRNAs binding to the rs1804197 and rs397768 loci and CRC risk. Second, the 3-year follow-up of CRC patients was relatively short. However, considering the high rate of becoming lost to follow-up after 5 years, we finally decided to select the data for 3 years of follow-up. In addition, genotype-based mRNA expression analysis was not performed in the present study, so further studies are needed to perform this.

## Conclusion

The rs1804197 locus in the 3′UTR region of the *APC* gene and its interaction with BMI and smoking are associated with the risk of CRC. Moreover, the interaction between SNP in the rs397768 locus and gender is associated with the risk of CRC in a Chinese Han population.

## Abbreviations

APC: adenomatous polyposis coli
ASD: autism spectrum disorder
BMI: body mass index
CI: confidence interval
CRC: colorectal cancer
FPRP: false positive report rate
MAF: minor allele frequency
MDR: multifactor dimensionality reduction
PFS: progression-free survival
SNP: single nucleotide polymorphism
UTR: untranslated region
